# The Effect of* Schinus terebinthifolius* Raddi (Anacardiaceae) Bark Extract on Histamine-Induced Paw Edema and Ileum Smooth Muscle Contraction

**DOI:** 10.1155/2017/1416375

**Published:** 2017-08-27

**Authors:** Paulo Alexandre Nunes-Neto, Tadeu José da Silva Peixoto-Sobrinho, Edilson Dantas da Silva Júnior, Jamilka Leopoldina da Silva, Alisson Rodrigo da Silva Oliveira, André Sampaio Pupo, Alice Valença Araújo, João Henrique da Costa-Silva, Almir Gonçalves Wanderley

**Affiliations:** ^1^Department of Pharmaceutical Sciences, Federal University of Pernambuco, Recife, PE, Brazil; ^2^Department of Pharmacology, Federal University of São Paulo, São Paulo, SP, Brazil; ^3^Department of Pharmacology, Instituto de Biociências, UNESP, Botucatu, São Paulo, SP, Brazil; ^4^Department of Physical Education and Sport Sciences, Federal University of Pernambuco, Vitória de Santo Antão, PE, Brazil; ^5^Department of Physiology and Pharmacology, Federal University of Pernambuco, Recife, PE, Brazil

## Abstract

*Schinus terebinthifolius* Raddi (Anacardiaceae), popularly known as red aroeira, is used in traditional medicine to treat inflammatory, gastric, and respiratory disorders. The aim of this study was to evaluate the antihistaminic activity of* S. terebinthifolius (St)* bark extract by using in vivo and in vitro experimental models. The effects of* St* were investigated on contractions induced by histamine, carbachol, and potassium chloride in isolated guinea pig ileum.* St* was also studied in response to hind paw edema induced by histamine in rats. Experiments revealed that although* St* (250, 500, and 1,000 *µ*g/mL) reduced the histamine-induced contractions by 9.1 ± 1.8, 50.2 ± 2.0, and 68.9 ± 2.0%, respectively, it did not inhibit contractions induced by carbachol or KCl. The association of* St* (250 and 500 *µ*g/mL) with hydroxyzine, an H_1_-antihistamine (0.125 and 0.250 *µ*M), increased the inhibitory effect to 67.0 ± 3.2 and 85.1 ± 2.1%, respectively. Moreover,* St* (100, 200, and 400 mg/kg) decreased paw edema from its peak by 33.9, 48.4, and 54.8%, respectively, whereas hydroxyzine (70 mg/kg) inhibited the peak edema by 56.5%. Altogether, the results suggest that the bark extract of* S. terebinthifolius* has an antihistaminic effect (H_1_).

## 1. Introduction

The increased prevalence of certain allergic diseases in several countries during the past few decades represents a major medical problem worldwide. Lifelong, progressive, and severe, such diseases are also associated with mortality and significant morbidity.

Allergies are common diseases that affect humans with diverse manifestations. They can induce several types of immune reactions, including type-I hypersensitivity reactions and the activation of mast cells [1, 2], the latter of which are key causes of allergic inflammation. Briefly, the release of histamine from activated mast cells and basophils contributes significantly to symptoms of rhinitis, sinusitis, conjunctivitis, urticaria, and other allergic reactions, including angioedema, asthma, anaphylaxis, and contact dermatitis [3].

Histamine is the most characterized and potent vasoactive mediator implicated in the acute phase of hypersensitivity. Its release can induce smooth muscle contraction, vasodilatation, and increased vascular permeability, among other conditions [4, 5]. Currently, four human G-protein-coupled histamine receptor subtypes (H_1–4_) are known to mediate the various actions of the monoamine. The histamine H_1_ receptor has been an attractive target for drug discovery for several years, and H_1_ receptor antagonists have proven to be effective therapeutic agents for allergy and respiratory disorders [6].

The Brazilian pepper tree, or read aroeira (*Schinus terebinthifolius* Raddi, Anacardiaceae), is a perennial plant native to Latin America and widely found in northeast Brazil [7]. In folk medicine, its aqueous bark extract is used to treat gastric ulcers [8], and it is also popularly used as an antiseptic, anti-inflammatory, balsamic, and hemostatic agent [9]. The importance of the plant has prompted its inclusion in Brazilian pharmacopoeia, in which the decoction of its bark is used as an anti-inflammatory agent [10].

The species has been frequently studied from a chemical viewpoint, and the presence of several constituents has been established, including phenols [11], pentagalloylglucose (i.e., a precursor of many complex structures of tannins) [12], and flavonoids [13, 14]. Chemical analysis of the bark of* S. terebinthifolius* has revealed the presence of anthraquinones, flavonoids, xanthones, saponins, pentacyclic triterpenes, and free steroids [15].

The increased incidence of allergic diseases in the human population has stimulated the search for bioactive products with potential antihistamine activity [16, 17]. In contribution, the aim of this study was to evaluate the antihistaminic activity of* S. terebinthifolius *bark extract by using in vivo and in vitro experimental models.

## 2. Material and Methods

### 2.1. Plant Material and Extraction

Bark from the stem of* S. terebinthifolius* Raddi was collected in the remains of the Atlantic rainforest located in the municipality of Cabo de Santo Agostinho, Pernambuco, Brazil (8° 20′ 33′′ S, 34° 56′ 59′′ W) in May 2013. A voucher specimen was authenticated in the Department of Botany at the Federal University of Pernambuco by curator M. Barbosa and deposited at Geraldo Mariz Herbarium (number 8758). Extraction was performed by the maceration of 250 g of pulverized* S. terebinthifolius *bark in 500 mL of 70% ethanol, at room temperature for 7 d. Crude ethanolic extract was filtered and evaporated under reduced pressure at 45°C for the complete elimination of the alcohol, followed by lyophilization, yielding approximately 17,4 g of dry residue. The lyophilized extract of* S. terebinthifolius* was kept at room temperature until use and suspended in distilled water.

### 2.2. High-Performance Liquid Chromatography Analysis

The chief phytochemical markers (i.e., gallic acid, ellagic acid, catechin, and epicatechin) in* S. terebinthifolius* samples were analyzed by liquid chromatography-diode array detection (LC-DAD) analysis using a HPLC system (LC-20AT, Shimadzu, Kyoto, Japan) equipped with a photodiode array detector (SPD-M20A, Shimadzu, Kyoto, Japan). Chromatographic separation was performed using a Gemini RP-18 column (5 *µ*m particle size and 250 × 4.60 mm i.d.; Phenomenex, Torrance, CA, USA) protected by a guard column of the same material.

Gradient elution was performed by varying the proportion of solvent A (0.5% acetic acid in distilled water, v/v) and solvent B (methanol) at a flow rate of 0.8 mL/min following a gradient program of 20–40% B (10 min), 40–60% B (10 min), 60% B (10 min), 60–40% B (10 min), and 40–20% B (10 min). The dried extracts and standards were dissolved in methanol: water (20 : 80, v/v) and filtered through a membrane of 0.45 *µ*m (Millipore, Billerica, MA, USA) prior to injection of 20 *µ*L. The peaks of each marking on dry substance were identified by comparing retention times and UV spectra of DAD.

### 2.3. Animals

Adult guinea pigs* (Cavia porcellus)* weighing 350–500 g and Wistar rats* (Rattus norvegicus)* weighing 250–300 g of both sexes, obtained from the Department of Physiology and Pharmacology at the Federal University of Pernambuco, were used for the in vitro and in vivo experiments, respectively. The animals had free access to standard food and water and were kept in separate rooms at 22 ± 2°C with 55–65% humidity with a 12 h light-dark cycle. All experimental protocols were submitted to and approved by the Animal Experimentation Ethics Committee of the Federal University of Pernambuco (license number 045543) in accordance with the National Institutes of Health's (Washington, DC, 2011)* Guide for the Care and Use of Laboratory Animals.*

### 2.4. Solutions and Drugs

In experiments, potassium chloride (KCl), histamine dihydrochloride, carbamylcholine chloride (carbachol), hydroxyzine dihydrochloride, verapamil hydrochloride, and 2-pyridylethylamine were manually diluted in distilled water and added to the organ bath. The nutrient solution used was modified Krebs solution with the composition (in mM) NaCl (117.0), NaHCO_3_ (25.0), NaH_2_PO_4_ (1.2), CaCl_2_ (2.5), KCl (4.7), MgSO_4_ (1.3), and glucose (11.0), pH = 7.4. Drugs were purchased from Sigma-Aldrich (St. Louis, MO, USA) and Vetec (Rio de Janeiro, RJ, Brazil).

### 2.5. Measurement of Ileum Contractile Activity

The experiment was performed by using the method described by Neto [16]. After fasting for 18 h, with water available ad libitum, the guinea pigs were euthanized by CO_2_ inhalation. The ileum was immediately removed, cleaned of connective tissue, and immersed in nutrition solution at room temperature. Roughly 2 cm long segments of the ileum were individually suspended in a 5 mL organ bath containing modified Krebs solution at 37°C and continuously gassed with a carbogenic mixture (95% O_2_ and 5% CO_2_). With the tissues oriented along their longitudinal axes, one side of the tissue was fixed to the bath and the other connected to a force transducer in order to measure isometric tension. The segments were allowed to stabilize for 30 min under a resting tension of 1 g. Thereafter, the preparations were washed every 10 min. The contractions were measured using force transducers coupled to an amplifier (AECAD 04F, AVS Projects, São Paulo, Brazil) connected to a computer with AQCAD 2.0.4 software (AVS Projects).

### 2.6. Experimental Protocols

#### 2.6.1. Effect of* S. terebinthifolius* Bark Extract on Contractions Induced by Histamine, Carbachol, and KCl

After the stabilization period, two submaximal contractions of the extract (selected from concentration-response curves) of similar amplitude were induced by an agonist of histamine receptors (histamine, 1 *μ*M), an agonist of muscarinic receptors (carbachol, 1 *μ*M), or a membrane depolarizing agent (KCl, 40 mM) at intervals of 30 min. The second contraction was defined as the control (100%). Thereafter the extract of* S. terebinthifolius* (250, 500, and 1,000 *μ*g/mL) was added to the bath; each preparation was exposed to only one concentration of the extract. After an incubation period of 20 min, a new contraction was induced by the contractile agent in the presence of the extract, and the amplitude of the contraction was measured.

#### 2.6.2. Effect of Hydroxyzine on Contractions Induced by Histamine, Carbachol, and KCl

After the appropriate stabilization of the preparations, the ileum was contracted two times with histamine (1 *μ*M), carbachol (1 *μ*M), or KCl (40 mM) at intervals of 30 min. The second contraction was defined as the control (100%). Then hydroxyzine (0.125 and 0.250 *μ*M), a H_1_-antihistamine, was added to the bath; each preparation was exposed to only one concentration of hydroxyzine. After an incubation period of 20 min, a new contraction was induced by the contractile agent in the presence of the H_1_-antihistamine.

#### 2.6.3. Effect of Verapamil on Contractions Induced by Histamine, Carbachol, and KCl

After the stabilization period, two contractions with similar amplitude were obtained by histamine (1 *μ*M), carbachol (1 *μ*M), or KCl (40 mM) at intervals of 30 min. The second contraction was defined as the control (100%). Once verapamil (0.350 and 0.700 *μ*M), a voltage-activated calcium channel blocker, was added to bath, each preparation was exposed to only one concentration of verapamil. After an incubation period of 20 min, a new contraction was induced by the contractile agent in the presence of verapamil.

#### 2.6.4. Effect of the Association of* S. terebinthifolius* Bark Extract and Hydroxyzine on Contractions Induced by Histamine

After the appropriate stabilization of the preparations, the ileum was contracted two times with histamine (1 *μ*M) at intervals of 30 min. The second contraction was defined as the control (100%). Once hydroxyzine plus* S. terebinthifolius* bark extract (0.125 + 250 and 0.250 *μ*M + 500 *μ*g/mL) was added to bath, each preparation was exposed to only one concentration of the mixture. After an incubation period of 20 min, a third contraction was induced by histamine.

#### 2.6.5. Effect of* S. terebinthifolius *Bark Extract, Hydroxyzine, or Their Association on the Concentration-Effect Curve to Histamine

Following the stabilization period, two contractions of similar amplitude were elicited by histamine (1 *μ*M) at intervals of 30 min. The second contraction was defined as the control (100%). The preparations were incubated with* S. terebinthifolius* bark extract (250, 500, and 1,000 *μ*g/mL), hydroxyzine (0.125 and 0.250 *μ*M), or hydroxyzine plus* S. terebinthifolius* bark extract (0.125 + 250 and 0.250 *μ*M + 500 *μ*g/mL). After an incubation period of 20 min, histamine was cumulatively added to bath (10^−9^ up to 10^−4^ M), and a concentration-effect curve was obtained.

#### 2.6.6. Effect of* S. terebinthifolius *Bark Extract on the Concentration-Effect Curve to 2-Pyridylethylamine

Following the stabilization period, two contractions with similar scope were elicited by the H_1_ selective agonist 2-pyridylethylamine (2-PEA) at intervals of 30 min. The second contraction was defined as the control (100%). Once the preparations were incubated with* S. terebinthifolius* bark extract (250, 500, and 1,000 *μ*g/mL), each was exposed to only one concentration of the extract. After an incubation period of 20 min, 2-pyridylethylamine was cumulatively added to bath (10^−8^–10^−4^ M), and a concentration-effect curve was obtained.

### 2.7. Histamine-Induced Paw Edema in Rats

Doses of the extract were based on the study of Cavalher–Machado et al. [12]. The experiment was performed by using the method of Winter et al. [18] with slight modifications. After 12 h of fasting, Wistar rats were randomly divided into five groups (*n* = 3/sex/group). The first group received drinking water (10 mL/kg p.o.), whereas the second was treated with hydroxyzine (70 mg/kg, p.o.). The other groups were orally treated with* S. terebinthifolius* bark extract (100, 200, and 400 mg/kg, per os, respectively). Approximately 1 h after the treatments, edema was induced by the administration of histamine (0.1% w/v, 0.1 mL) in the right-hind paw's subplantar region. The results were obtained by the paw's volume difference before and after 0.5, 1, 2, 3, and 4 h of edema induction. Paw volume was measured by plethysmography (Model 7140, Ugo Basile, Varese, Italy).

### 2.8. Statistical Analysis

Results were expressed as mean ± SEM. Differences between means were determined using one-way analysis of variance followed by Tukey's multiple comparisons test. The level of significance for rejection of the null hypothesis was 5% (*p* < 0.05). The concentration of a substance that reduces the response to an agonist by 50% (IC_50_) was obtained by nonlinear regression. The maximum effect and negative logarithm of the molar concentration of an agonist that produces 50% of its maximal effect (pEC_50_) were obtained graphically from the concentration-response curve. Analyses were performed using GraphPad Prism 6.0 (GraphPad Software, Inc., La Jolla, CA, USA).

## 3. **Results**

### 3.1. Phytochemical Screening of* S. terebinthifolius* Bark Extract

**Figure 1:**
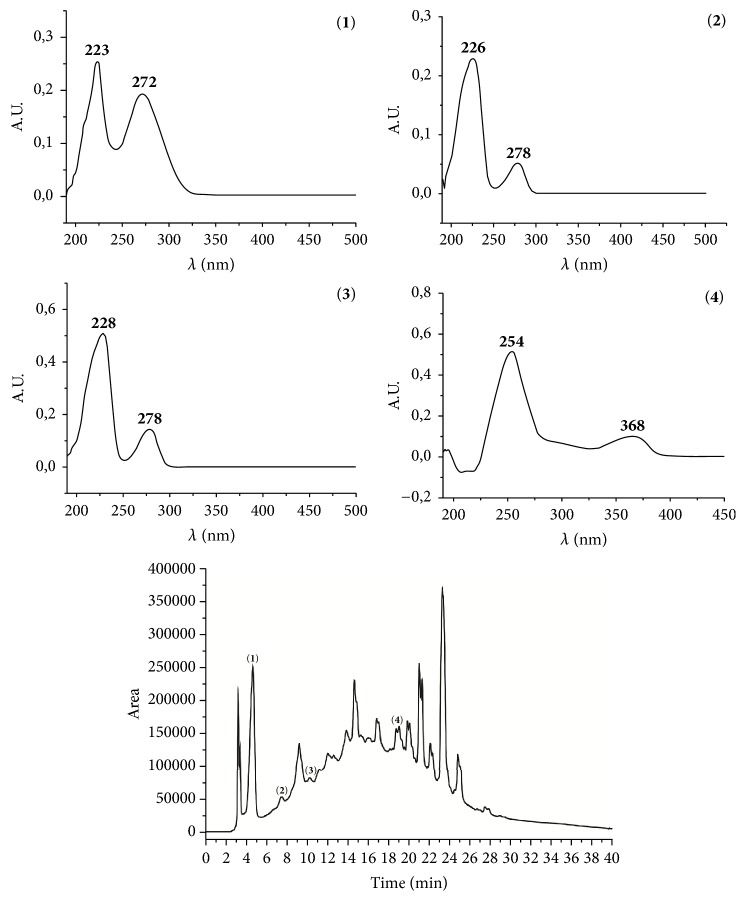
Chromatogram of* Schinus terebinthifolius (St)* bark extract detected at 278 nm. Peaks: (1) gallic acid (Rt = 4.66), (2) catechin (Rt = 7.51), (3) epicatechin (Rt = 10.24), and (4) ellagic acid (Rt = 19.05).

The LC-DAD for the chemical identification of the constituents of* S. terebinthifolius* bark extract revealed the presence of chromatographic peaks consistent with the standards. The retention times were 4.66, 7.51, 10.24, and 19.05 min for gallic acid, catechin, epicatechin, and ellagic acid, respectively (Figure 1).

### 3.2. Effect of* S. terebinthifolius* Bark Extract on Contractions Induced by Histamine, Carbachol, and KCl

**Figure 2:**
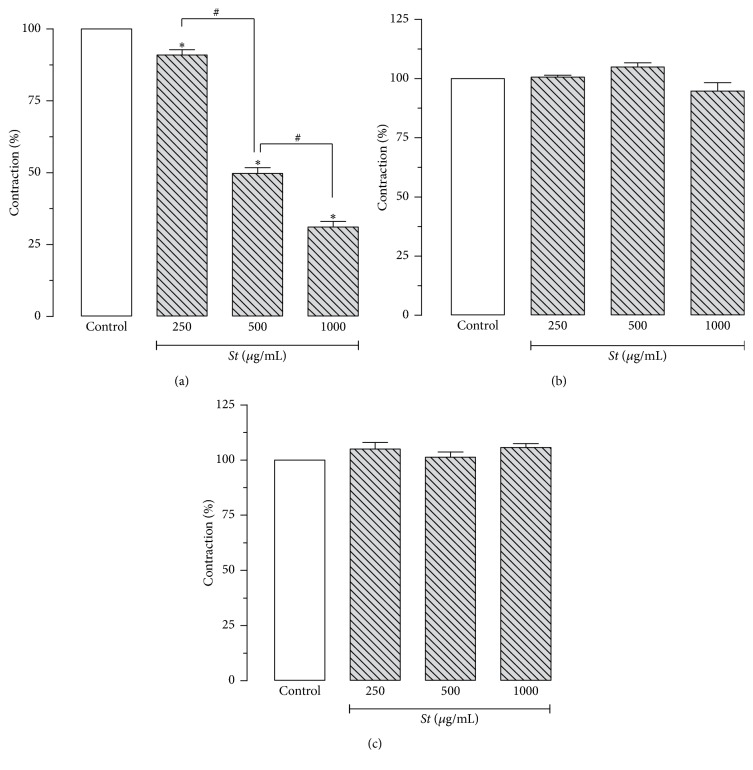
Effect of* Schinus terebinthifolius (St)* bark extract on the contractions induced by histamine (1 *μ*M; (a)), carbachol (1 *μ*M; (b)), and KCl (40 mM; (c)) in isolated guinea pig ileum. Columns and vertical bars represent the mean ± SEM, respectively (*n* = 6). ^*∗*^Statistically different from control. ^#^Significant difference (one-way ANOVA followed by Tukey's multiple comparisons test, *p* < 0.05).

*S. terebinthifolius* selectively inhibited contractions induced by histamine (1 *μ*M) in guinea pig ileum in a concentration-dependent manner. The reductions were 9.1 ± 1.8, 50.2 ± 2.0, and 68.9 ± 2.0% (*p* < 0.05) with doses of 250, 500, and 1,000 *μ*g/mL of the extract, respectively (*n* = 6). IC_50_ (95% CI) was 487.5 *μ*g/mL (460.3–517.6 *μ*g/mL). By contrast, the extract at the same concentrations showed no effect on carbachol- or KCl-induced contractions (Figure 2).

### 3.3. Effect of Hydroxyzine on Contractions Induced by Histamine, Carbachol, and KCl

Hydroxyzine decreased the amplitude of histamine-induced contractions by 25.9 ± 3.1 and 51.2 ± 3.0% (*p* < 0.05) in concentrations of 0.125 and 0.250 *μ*M, respectively (*n* = 6). Similar to the extract, hydroxyzine (0.250 *μ*M) showed no effect on the carbachol- or KCl-induced contractions. The percentage of contractions induced by carbachol and KCl was 100.4 ± 2.1 and 102.3 ± 1.6%, respectively.

### 3.4. Effect of Verapamil on Contractions Induced by Histamine, Carbachol, and KCl

Verapamil (0.350 *μ*M) decreased the amplitude of contractions induced by histamine and carbachol by 52.0 ± 4.3 and 54.8 ± 2.3% (*p* < 0.05), respectively, whereas contractions elicited with KCl reduced by 56.3 ± 2.9% (*p* < 0.05) in a concentration of 0.700 *μ*M (*n* = 6).

### 3.5. Effect of the Association of* S. terebinthifolius* Bark Extract and Hydroxyzine on Simple Contractions Induced by Histamine

**Figure 3:**
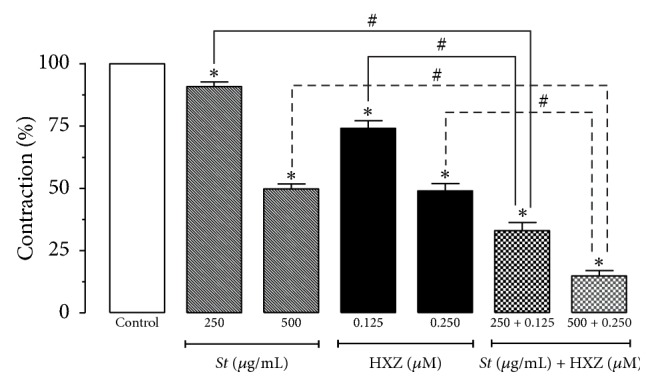
Effect of* Schinus terebinthifolius (St)* bark extract, hydroxyzine (HXZ), and* St* + HXZ on the contractions induced by histamine (1 *μ*M) in isolated guinea pig ileum. Columns and vertical bars represent the mean ± SEM, respectively (*n* = 6). ^*∗*^Statistically different from control. ^#^Significant difference (one-way ANOVA followed by Tukey's multiple comparisons test, *p* < 0.05).

The association of* S. terebinthifolius* and hydroxyzine induced an increase in the inhibitory effect of histamine-induced contractions compared to the inhibition produced by the extract or hydroxyzine alone. Reductions of 67.0 ± 3.2% and 85.1 ± 2.1% (*p* < 0.05) occurred with 250 + 0.125 and 500 *μ*g/mL + 0.250 *μ*M of* S. terebinthifolius* plus hydroxyzine, respectively (*n* = 6), as shown in Figure 3.

### 3.6. Effect of* S. terebinthifolius *Bark Extract, Hydroxyzine, or Their Association on the Concentration-Effect Curve to Histamine

**Figure 4:**
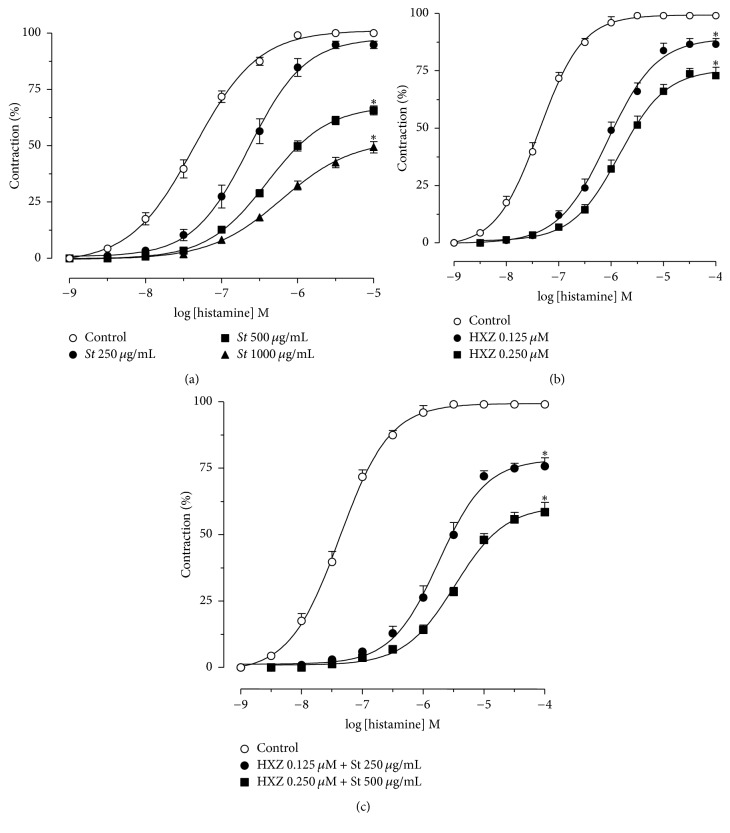
Cumulative concentration-effect curves to histamine in the absence (O) or in the presence of* Schinus terebinthifolius* bark extract (*St*, (a)): 250 (●), 500 (■), and 1.000 *μ*g/mL (▲); hydroxyzine (HXZ, (b)): 0.125 (●) and 0.250 *μ*M (■); and HXZ +* St* (c): 250 + 0.125 (●) and 500 *μ*g/mL + 0.250 *μ*M (■). Symbols and vertical bars represent the mean ± SEM, respectively (*n* = 6). ^*∗*^Statistically different from control (one-way ANOVA followed by Tukey's multiple comparisons test, *p* < 0.05).

At concentrations of 250, 500, and 1,000 *μ*g/mL,* S. terebinthifolius* rightward shifted histamine curves compared to the control 7.3, 9.7, and 11.6 times, respectively. The two highest concentrations of the extract (Figure 4(a)) also reduced the maximum effect (*E*_max_) of histamine. Hydroxyzine also induced a shift to the right of the concentration-effect curves of histamine, with a decrease in maximal effect. The shift was 13.0 and 15.2 times that of the control in the presence of 0.125 and 0.250 *μ*M of hydroxyzine, respectively (Figure 4(b)). The effects were even greater when the extract was associated with hydroxyzine, which shifted the curves to the right 16.2 and 18.9 times in concentrations of 250 + 0.125 and 500 *μ*g/mL + 0.250 *μ*M of* S. terebinthifolius* plus hydroxyzine, respectively (Figure 4(c)). Results in pharmacological parameters *E*_max_ and pEC_50_ appear in Table 1, which shows that the* S. terebinthifolius* (500 *μ*g/mL), hydroxyzine (0.250 *μ*M), and* S. terebinthifolius* plus hydroxyzine (500 *μ*g/mL + 0.250 *μ*M) groups statistically differed. However, statistical significance was not verified for the smaller doses and their association.

### 3.7. Effect of* S. terebinthifolius *Bark Extract on the Concentration-Effect Curve to 2-Pyridylethylamine

**Figure 5:**
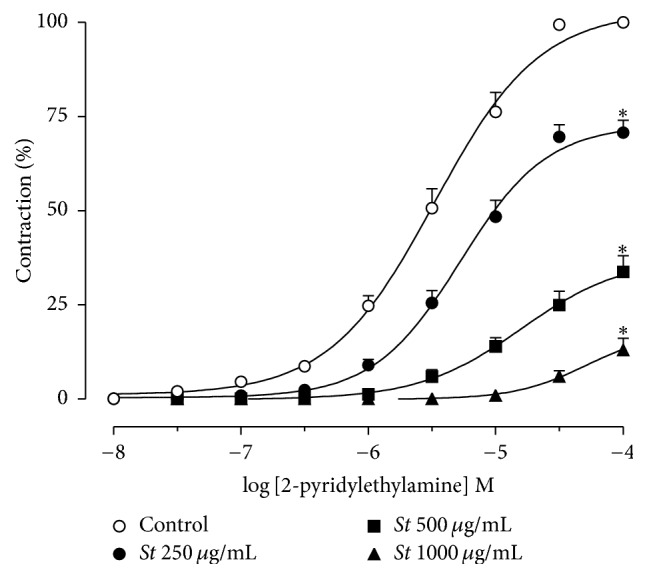
Cumulative concentration-effect curves to 2-pyridylethylamine in the absence (O) or in the presence of* Schinus terebinthifolius (St)* bark extract: 250 (●), 500 (■), and 1,000 *μ*g/mL (▲). Symbols and vertical bars represent the mean ± SEM, respectively (*n* = 8). ^*∗*^Statistically different from control (one-way ANOVA followed by Tukey's multiple comparisons test, *p* < 0.05).

*S. terebinthifolius *extract rightward shifted the concentration-effect curves for 2-PEA in a concentration-dependent manner compared to the control, with a maximum effect reduction for all concentrations of the extract (Figure 5). In the presence of 250, 500, and 1,000 *μ*g/mL of the extract, the shift was 2.1, 7.1, and 13.0 times (pEC_50_ = 5.25 ± 0.05; 4.79 ± 0.11 and 4.28 ± 0.12, respectively) that of the control (pEC_50_ = 5.45 ± 0.04), and *E*_max_ reduced from 99.4 ± 0.6% (control) to 70.7 ± 3.3, 35.6 ± 4.3, and 17.1 ± 2.9% (*p* < 0.05), respectively.

### 3.8. Histamine-Induced Paw Edema in Rats

Table 2 shows the effect of* S. terebinthifolius* on acute paw edema induced by histamine in rats. The maximum phlogistic response of histamine was observed 1 h after the injection of histamine. The extract caused a dose-dependent decrease of edema 1 h after its induction compared to the control group. This effect was monitored for another 3 h, during which time inhibition peaked at 53.7, 70.7, and 95.1% (*p* < 0.05) in animals treated with 100, 200, and 400 mg/kg of extract, respectively. The extract in a dose of 400 mg/kg and hydroxyzine (i.e., positive control) exhibited equivalent effects in the reduction of edema since those values were not statistically different.

## 4. **Discussion**

Many plant species used in folk medicine induce antihistaminic effects by inhibiting histamine release from mast cells or by blocking the H_1_ receptor [19–21]. Histamine plays a key role in many physiological processes, and drugs targeting H_1_ receptors have been used successfully [22, 23]. In this study, the possible antihistamine effect of* S. terebinthifolius* extract on H_1_ receptors was evaluated.

Ileum smooth muscle contraction is achieved by complex mechanisms related to a cascade of events involving several mediators. Together, they culminate in increasing intracellular calcium concentration [Ca^2+^]_i_ as well, which activates the contraction mechanism. The increase in [Ca^2+^]_i_ can be directly induced by plasma membrane depolarization by depolarizing agents via electromechanical coupling, including KCl, which induces calcium influx through voltage-activated calcium channels (VACCs). Otherwise, it can be induced by a receptor agonist via pharmacomechanical coupling. Agonists such as histamine and carbachol bind to G-protein-coupled receptors (*G*_q/11_ protein activation) and activate the phosphoinositide cascade, thereby mediating the production of inositol 1,4,5-trisphosphate (IP_3_), which stimulates calcium release from the sarcoplasmic reticulum, and diacylglycerol, which, along with calcium, activates the same protein kinase C that phosphorylates L-type VACCs, resulting in calcium influx and depolarization. Contractile agents can also increase [Ca^2+^]_i_ through the ryanodine receptor or the induction of calcium influx across the membrane through various classes of calcium channels [24–26].

Results shown in Figure 2 reveal that* S. terebinthifolius* extract selectively inhibited histamine-induced contractions in a concentration-dependent manner, but it did not exert any effect on carbachol- or KCl-induced contractions in guinea pig ileum. It is therefore reasonable to propose that the extract may act directly on the histaminergic receptor or in its signaling pathway, since the effect on the muscarinic (M_3_) and Ca^2+^ influx through VACCs was not altered by the presence of the extract. Similarly, hydroxyzine, a standard H_1_-antihistamine, also caused the inhibition of contractions induced by histamine but not carbachol or KCl (Figure 3). Thus,* S. terebinthifolius* extract exhibits a similar inhibitory profile of hydroxyzine on the contractile activity in guinea pig ileum, which suggests an antihistamine effect possibly due to an action on H_1_ receptors.

First-generation H_1_-antihistamines are known to interact with receptors other than H_1_, including muscarinic receptors [22]. However, in this study, an inhibitory effect of hydroxyzine on contractions induced by carbachol on guinea pig ileum was not observed, which suggests that hydroxyzine in that concentration does not interfere with the activation of muscarinic receptors.

The activation of calcium channels is a common mechanism involved in contractions induced by histamine, carbachol, and KCl. As expected, verapamil, a VACCs blocker, inhibited contractions induced by the three agents. Such results indicate that the inhibition caused by* S. terebinthifolius* extract does not occur on a common cellular target such as the VACCs but could be due to a direct action on the H_1_ receptor, given that contractions induced by carbachol or KCl did not change in the presence of the extract.

The antihistaminic effect of* S. terebinthifolius* extract was also evaluated in association with hydroxyzine (250 *µ*g/mL + 0.125 *µ*M or 500 *µ*g/mL + 0.250 *µ*M *St* + HXZ), to verify a possible synergic effect (Figure 3). When the drugs were used simultaneously in both concentrations, an increase in inhibition occurred that was greater than that of their administration alone, which suggests a synergic effect.

Gaddum [27] described a useful empirical classification of receptor antagonisms as surmountable and insurmountable antagonisms. A surmountable antagonist produces dextral displacement (i.e., shifts to the right) of agonist concentration-response curves with no concomitant decrease of the maximal response to the agonist. The standard method of determining the potency of such an antagonist is Schild analysis or with a Clark plot. A prerequisite of the correct use of those methods is the demonstration of the parallel displacement of the agonist concentration-response with no reduction of maximal response to the agonist. By contrast, insurmountable antagonists depress the maximal response, and the determination of potency depends on the model of antagonism used for data comparison [28]. The most common explanations for insurmountable antagonisms are the slow dissociation of the antagonist-receptor complex and the binding of the antagonist to the allosteric site of the receptor [29].

At a lower dose (250 *µ*g/mL), the extract induced a parallel shift to the right of the concentration-effect curve to histamine, thereby suggesting a surmountable antagonism. However, at higher doses (500 and 1,000 *µ*g/mL), the shift to the right with a decrease of the maximal effect implied an insurmountable antagonistic profile.

Interestingly, the pattern of the curve (i.e., shift to the right, with a decrease of the maximal effect) was similar to that observed for hydroxyzine. The decrease in the pharmacological parameters *E*_max_ and pEC_50_ was even greater when the extract was associated with hydroxyzine. Such results suggest an insurmountable antagonism, since the maximal effect of the concentration-effect curves in the presence of the extract was reduced.

However, the histamine receptors also presented* constitutive activity*, defined as the ability to trigger downstream events even in the absence of a ligand binding. Their active and inactive states exist in equilibrium; at rest, the inactive state isomerizes with the active state and vice versa. H_1_-antihistamines act as inverse agonists that combine with and stabilize the inactive conformation of the H_1_-receptor, thereby shifting the equilibrium toward the inactive state. For more than 50 years, they were described as H_1_-receptor antagonists or H_1_-receptor blockers; however, those out-of-date terms do not accurately reflect their molecular mechanism of action [30].

To confirm the action of* S. terebinthifolius* extract on H_1_ receptors and eliminate the effect of histamine on other subtypes of histamine receptors present in the guinea pig ileum, concentration-effect curves to a H_1_ selective agonist, 2-pyridylethylamine, was performed in the absence and presence of the extract. Results showed a decrease in the potency of 2-pyridylethylamine and a reduction of the maximum effect in the presence of the extract at all concentrations studied, which are characteristic of insurmountable antagonisms.

The antihistaminic activity of* S. terebinthifolius* extract was also demonstrated in an in vivo assay via the inhibition of paw edema induced by histamine in rats. Histamine induces edema primarily through its action on H_1_ receptors in vascular tissue. In response to that interaction, vasodilatation and increased vascular permeability occur, thereby allowing the extravasation of plasma proteins and intravascular fluid into the interstitium, with edema formation as a result. In local inflammatory response, polymorphonuclear leukocyte infiltration and cytokine release occur [31, 32]. Results of the present study show that the extract prevented the edematogenic effects produced by histamine, which reinforces the assumption of the action on the H_1_ receptor. From those data, it is possible to infer that the H_1_-antihistamine effect of the extract could be useful against allergic processes triggered by the primary mediator histamine. The leaves of* S. terebinthifolius* have been shown to present antiallergic properties in animal models [12]. Previous studies have shown that acute and subacute (45-d) oral administration of* S. terebinthifolius* bark extract (0.25, 0.625, and 1.5625 g/kg) did not induce any toxic effects in Wistar rats, which indicates that the extract can be safely administered orally [33].

Based on the results obtained with* S. terebinthifolius* extract and considering (i) the selective decrease in histamine-induced contractions in guinea pig ileum and lack of effect on the magnitude of contractions induced by carbachol and KCl, (ii) the inhibitory effect similar to that exerted by hydroxyzine (H_1_-antihistamine) but different from that observed with the VACC verapamil on contractions induced by histamine, carbachol, and KCl, (iii) the rightward displacement of all concentration-effect curves for histamine in a concentration-dependent manner and the potentiation of the antihistaminic effect of hydroxyzine in combination with the extract, (iv) the rightward displacement of all concentration-effect curves for 2-pyridylethylamine (histamine H_1_-receptor agonist) in a concentration-dependent manner, and (v) the decrease in histamine-induced paw edema in rats, results suggest that* S. terebinthifolius* bark extract has antihistaminic activity and that its probable action mechanism is a direct interaction with histamine H_1_ receptors, which constitutes an insurmountable antagonism.

## Figures and Tables

**Table 1:** *E*_max_ and pEC_50_ values of concentration-response curves to histamine in the absence and presence of *Schinus terebinthifolius (St) *bark extract, hydroxyzine (HXZ), and their associations.

Treatment	*E*_max_ (%)	pEC_50_
Control	99.0 ± 1.4	7.37 ± 0.03
*St* 250 *μ*g/mL	94.7 ± 1.6	6.64 ± 0.05^*∗*^
*St* 500 *μ*g/mL	65.7 ± 1.9^*∗*^	6.40 ± 0.04^*∗*,#^
*St* 1,000 *μ*g/mL	49.4 ± 2.5^*∗*^	6.21 ± 0.07^*∗*^
HXZ 0.125 *μ*M	86.6 ± 2.5^*∗*^	6.07 ± 0.05^*∗*^
HXZ 0.250 *μ*M	72.9 ± 3.6^*∗*^	5.85 ± 0.06^*∗*,#^
*St* 250 *μ*g/mL + HXZ 0.125 *μ*M	75.7 ± 3.2^*∗*^	5.75 ± 0.05^*∗*^
*St* 500 *μ*g/mL + HXZ 0.250 *μ*M	58.4 ± 3.7^*∗*^	5.48 ± 0.05^*∗*,#^

Values represent the mean ± SEM (*n* = 6). ^*∗*^Statistically different compared to the control. ^#^The groups are statistically different from each other. (One-way ANOVA followed by Tukey's multiple comparisons test, *p* < 0.05.)

**Table 2:** Effect of the oral administration of *Schinus terebinthifolius (St)* bark extract on paw edema induced by histamine in Wistar rats.

Groups	Dose (mg/kg)	Edema volume (mL) at different time interval				
		0.5 h	1 h	2 h	3 h	4 h
Control	—	0.26 ± 0.02	0.62 ± 0.03	0.47 ± 0.02	0.44 ± 0.03	0.41 ± 0.03
HXZ	70	0.20 ± 0.01	0.27 ± 0.01	0.15 ± 0.02	0.10 ± 0.02	0.04 ± 0.01
		(23.1%)	(56.5%)^*∗*^	(68.1%)^*∗*^	(77.3%)^*∗*^	(90,2%)^*∗*^
*St*	100	0.21 ± 0.02	0.41 ± 0.02	0.31 ± 0.02	0.24 ± 0.03	0.19 ± 0.04
		(19.2%)	(33.9%)^*∗*^	(34.0%)^*∗*^	(45.5%)^*∗*^	(53.7%)^*∗*^
*St*	200	0.22 ± 0.02	0.32 ± 0.03	0.24 ± 0.03	0.18 ± 0.02	0.12 ± 0.02
		(15.4%)	(48.4%)^*∗*^	(48.9%)^*∗*^	(59.1%)^*∗*^	(70.7%)^*∗*^
*St*	400	0.18 ± 0.03	0.28 ± 0.01	0.17 ± 0.02	0.06 ± 0.01	0.02 ± 0.01
		(30.8%)	(54.8%)^*∗*^	(63.8%)^*∗*^	(86.4%)^*∗*^	(95.1%)^*∗*^

Values represent the mean ± SEM (*n* = 3/sex/group). HXZ: hydroxyzine. ^*∗*^Statistically different compared to the control. (One-way ANOVA followed by Tukey's multiple comparisons test, *p* < 0.05.)
